# 6-Ferrocenoyl-7-phenyl­spiro­[hexa­hydro­pyrrolo­[1,2-*c*][1,3]thia­zole-5,11′-indeno­[1,2-*b*]quinoxaline]

**DOI:** 10.1107/S1600536813022605

**Published:** 2013-08-17

**Authors:** Sivasubramanian Suhitha, Krishnaswamy Gunasekaran, Adukamparai Rajukrishnan Sureshbabu, Raghavachary Raghunathan, Devadasan Velmurugan

**Affiliations:** a Centre of Advanced Study in Crystallography and Biophysics, University of Madras, Guindy Campus, Chennai 600 025, India; b Department of Organic Chemistry, University of Madras, Guindy Campus, Chennai 600 025, India

## Abstract

In the title compound, [Fe(C_5_H_5_)(C_32_H_24_N_3_OS)], both the thia­zolidine ring and the pyrrolidine ring adopt an envelope conformation, with the S atom and the phenyl-bearing C atom, respectively, as the flaps. The thia­zolidine ring mean plane makes a dihedral angle of 59.08 (11)° with the pyrrolidine ring mean plane, which in turn makes a dihedral angle of 83.40 (10)° with the cyclo­pentane ring, indicating that the latter two rings are almost orthogonal to one another. In the crystal, a pair of C—H⋯O hydrogen bonds link the mol­ecules forming inversion dimers. The dimers are linked *via* π–π inter­actions [centroid–centroid distance = 3.7764 (10) Å] involving the quinoxaline moieties forming chains propagating along [1-10].

## Related literature

For the biological activity of ferrocene derivatives, see: Jaouen *et al.* (2004 ▶); Biot *et al.* (2004 ▶); Fouda *et al.* (2007 ▶). For a related structure, see: Vijayakumar *et al.* (2012 ▶).
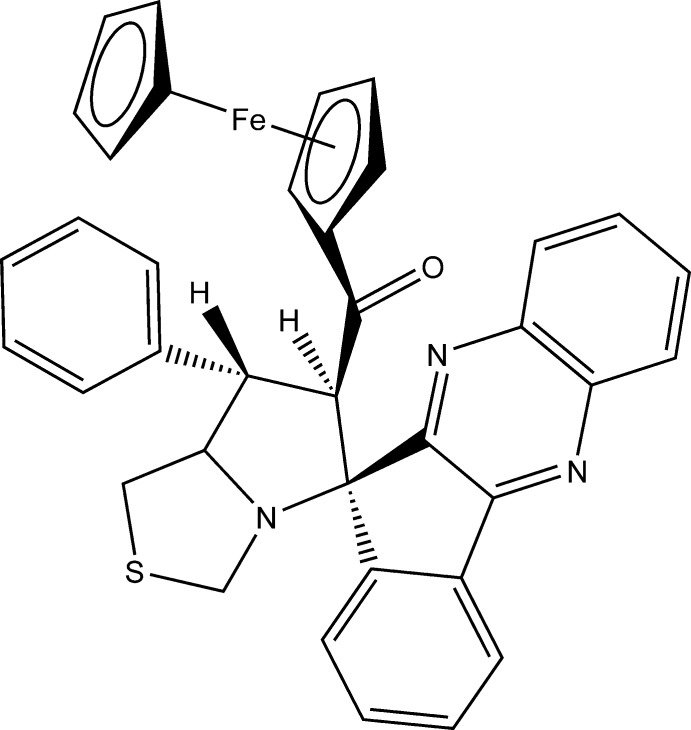


## Experimental

### 

#### Crystal data

[Fe(C_5_H_5_)(C_32_H_24_N_3_OS)]
*M* *_r_* = 619.55
Triclinic, 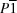

*a* = 9.5687 (3) Å
*b* = 10.0175 (3) Å
*c* = 17.1768 (5) Å
α = 80.367 (2)°
β = 75.430 (1)°
γ = 65.293 (1)°
*V* = 1444.02 (8) Å^3^
*Z* = 2
Mo *K*α radiation
μ = 0.63 mm^−1^
*T* = 293 K
0.30 × 0.25 × 0.20 mm

#### Data collection

Bruker SMART APEXII area-detector diffractometer
Absorption correction: multi-scan (*SADABS*; Bruker, 2008 ▶) *T*_min_ = 0.833, *T*_max_ = 0.884
21307 measured reflections
5884 independent reflections
5158 reflections with *I* > 2σ(*I*)
*R*_int_ = 0.025

#### Refinement

*R*[*F* ^2^ > 2σ(*F* ^2^)] = 0.034
*wR*(*F* ^2^) = 0.094
*S* = 1.02
5884 reflections
388 parameters
H-atom parameters constrained
Δρ_max_ = 0.30 e Å^−3^
Δρ_min_ = −0.33 e Å^−3^

### 

Data collection: *APEX2* (Bruker, 2008 ▶); cell refinement: *SAINT* (Bruker, 2008 ▶); data reduction: *SAINT*; program(s) used to solve structure: *SHELXS97* (Sheldrick, 2008 ▶); program(s) used to refine structure: *SHELXL97* (Sheldrick, 2008 ▶); molecular graphics: *ORTEP-3 for Windows* (Farrugia, 2012 ▶); software used to prepare material for publication: *SHELXL97* and *PLATON* (Spek, 2009 ▶).

## Supplementary Material

Crystal structure: contains datablock(s) global, I. DOI: 10.1107/S1600536813022605/su2630sup1.cif

Structure factors: contains datablock(s) I. DOI: 10.1107/S1600536813022605/su2630Isup2.hkl

Additional supplementary materials:  crystallographic information; 3D view; checkCIF report

## Figures and Tables

**Table 1:** Hydrogen-bond geometry (Å, °)

*D*—H⋯*A*	*D*—H	H⋯*A*	*D*⋯*A*	*D*—H⋯*A*
C25—H25⋯O1^i^	0.93	2.58	3.371 (3)	143

Symmetry code: (i) 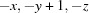.

**Fig. 1.**
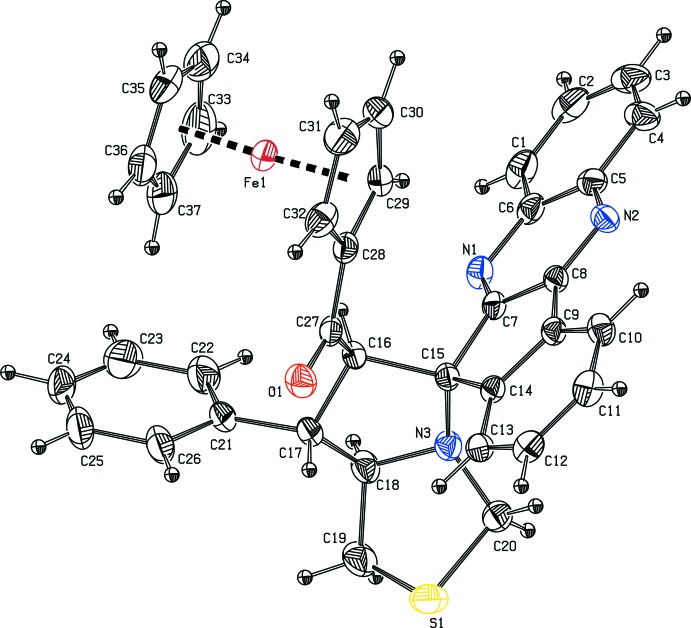
The molecular structure of the title molecule, with atom labelling. Displacement ellipsoids are drawn at the 30% probability level.

**Fig. 2.**
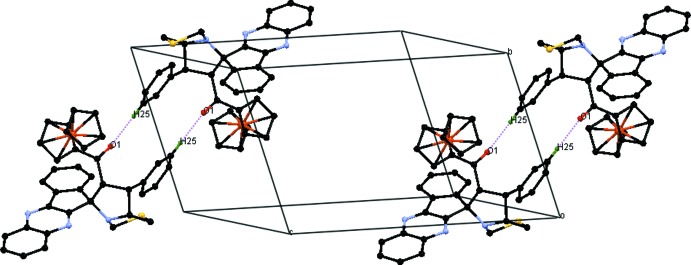
The crystal packing of the title compound viewed along the a axis. The C-H···O hydrogen bonds are shown as dashed lines (see Table 1 for details; H-atoms not involved in hydrogen bonding have been omitted for clarity).

## supplementary crystallographic information

### 1. Comment

Ferrocene attached compounds are well known to have biological activities, such as antimalarial and antifungal (Biot *et al.*, 2004), antitumor (Jaouen *et al.*, 2004), and antibacterial (Fouda *et al.*, 2007). Against this background, and in order to obtain information on the molecular conformation of such compounds, we synthesised the title compound and report herein on its crystal structure.

In the title compound, Fig. 1, both the thiazolidine ring (A = S1/N3/C18-C20) and the pyrrolidine ring (B = N3/C15-C18) adopt *envelope* conformations with atoms S1 and C17, respectively, as the flaps. Their mean planes, A and B, make a dihedral angle of 59.08 (11)°. The cyclopentane ring (C = C7-C9/C14/C15) mean plane is inclined to the thiazolidine and pyrrolidine ring planes by C/A = 38.89 (10)° and C/B = 83.40 (10)°, indicating that the latter two rings are almost orthogonal to each other.

In the crystal, a pair of C—H···O hydrogen bonds link the molecules forming inversion dimers (Table 1 and Fig. 2). The dimers are linked via π-π interactions involving the quinoxaline moieties forming chains propagating along [1-10]; Cg1···Cg2^i^ = 3.7764 (10) Å [Cg1 and Cg2 are the centroids of rings (N1/N2/C5-C8) and (C9-C14), respectively; symmetry code: (i) = -x, -y, -z+1].

### 2. Experimental

Ninhydrin (1 mmol) and 1, 2-phenylenediamine (1 mmol) were mixed and stirred with 10 ml of methanol for 10 min. To this mixture 1 mmol of thioproline and 1 mmol of Ferrocene derived dipolarophile were added and the mixture was refluxed up to the end of the reaction as observed by TLC. The solvent was removed under reduced pressure and the crude product obtained was purified by column chromatography, using a 4:1 ratio of petroleum ether and ethyl acetate. Block-like colourless crystals suitable for the X-ray diffraction were obtained by slow evaporation of the solvent at room temperature.

### 3. Refinement

Hydrogen atoms were placed in calculated positions with C—H ranging from 0.93Å to 0.98Å and refined using the riding model approximation with U_iso_(H) = 1.2U_eq_(C).

### Crystal data

**Table:**

[Fe(C_5_H_5_)(C_32_H_24_N_3_OS)]	*Z* = 2
*M**_r_* = 619.55	*F*(000) = 644
Triclinic, *P*1	*D*_x_ = 1.425 Mg m^−^^3^
Hall symbol: -P 1	Mo *K*α radiation, λ = 0.71073 Å
*a* = 9.5687 (3) Å	Cell parameters from 5884 reflections
*b* = 10.0175 (3) Å	θ = 1.2–26.4°
*c* = 17.1768 (5) Å	µ = 0.63 mm^−^^1^
α = 80.367 (2)°	*T* = 293 K
β = 75.430 (1)°	Block, colourless
γ = 65.293 (1)°	0.30 × 0.25 × 0.20 mm
*V* = 1444.02 (8) Å^3^	

### Data collection

**Table:**

Bruker SMART APEXII area-detector diffractometer	5884 independent reflections
Radiation source: fine-focus sealed tube	5158 reflections with *I* > 2σ(*I*)
Graphite monochromator	*R*_int_ = 0.025
ω and φ scans	θ_max_ = 26.4°, θ_min_ = 1.2°
Absorption correction: multi-scan (*SADABS*; Bruker, 2008)	*h* = −11→11
*T*_min_ = 0.833, *T*_max_ = 0.884	*k* = −12→12
21307 measured reflections	*l* = −21→21

### Refinement

**Table:**

Refinement on *F*^2^	Primary atom site location: structure-invariant direct methods
Least-squares matrix: full	Secondary atom site location: difference Fourier map
*R*[*F*^2^ > 2σ(*F*^2^)] = 0.034	Hydrogen site location: inferred from neighbouring sites
*wR*(*F*^2^) = 0.094	H-atom parameters constrained
*S* = 1.02	*w* = 1/[σ^2^(*F*_o_^2^) + (0.0468*P*)^2^ + 0.5453*P*] where *P* = (*F*_o_^2^ + 2*F*_c_^2^)/3
5884 reflections	(Δ/σ)_max_ < 0.001
388 parameters	Δρ_max_ = 0.30 e Å^−^^3^
0 restraints	Δρ_min_ = −0.33 e Å^−^^3^

### Special details

**Table:**

Geometry. All esds (except the esd in the dihedral angle between two l.s. planes) are estimated using the full covariance matrix. The cell esds are taken into account individually in the estimation of esds in distances, angles and torsion angles; correlations between esds in cell parameters are only used when they are defined by crystal symmetry. An approximate (isotropic) treatment of cell esds is used for estimating esds involving l.s. planes.
Refinement. Refinement of F^2^ against ALL reflections. The weighted R-factor wR and goodness of fit S are based on F^2^, conventional R-factors R are based on F, with F set to zero for negative F^2^. The threshold expression of F^2^ > 2sigma(F^2^) is used only for calculating R-factors(gt) etc. and is not relevant to the choice of reflections for refinement. R-factors based on F^2^ are statistically about twice as large as those based on F, and R- factors based on ALL data will be even larger.

### Fractional atomic coordinates and isotropic or equivalent isotropic displacement parameters (Å^2^)

**Table:**

	*x*	*y*	*z*	*U*_iso_*/*U*_eq_	
C1	0.5950 (2)	−0.2678 (2)	0.33088 (13)	0.0572 (5)	
H1	0.6174	−0.3016	0.2801	0.069*	
C2	0.7052 (2)	−0.3259 (2)	0.37816 (16)	0.0640 (6)	
H2	0.8026	−0.3984	0.3590	0.077*	
C3	0.6729 (2)	−0.2776 (2)	0.45501 (16)	0.0625 (6)	
H3	0.7493	−0.3181	0.4863	0.075*	
C4	0.5309 (2)	−0.1717 (2)	0.48453 (14)	0.0538 (5)	
H4	0.5104	−0.1414	0.5360	0.065*	
C5	0.4146 (2)	−0.10804 (18)	0.43704 (11)	0.0413 (4)	
C6	0.44732 (19)	−0.15649 (18)	0.35905 (11)	0.0424 (4)	
C7	0.20582 (18)	0.00231 (16)	0.34185 (9)	0.0337 (3)	
C8	0.17255 (18)	0.05027 (16)	0.42025 (9)	0.0333 (3)	
C9	0.01188 (18)	0.16204 (16)	0.43519 (9)	0.0341 (3)	
C10	−0.0740 (2)	0.24182 (18)	0.50220 (10)	0.0423 (4)	
H10	−0.0295	0.2306	0.5464	0.051*	
C11	−0.2280 (2)	0.33872 (19)	0.50161 (11)	0.0467 (4)	
H11	−0.2880	0.3928	0.5461	0.056*	
C12	−0.2934 (2)	0.35576 (18)	0.43558 (11)	0.0442 (4)	
H12	−0.3974	0.4204	0.4367	0.053*	
C13	−0.20732 (19)	0.27862 (17)	0.36781 (10)	0.0387 (3)	
H13	−0.2517	0.2923	0.3233	0.046*	
C14	−0.05306 (18)	0.18035 (16)	0.36777 (9)	0.0328 (3)	
C15	0.06564 (18)	0.08425 (16)	0.30108 (9)	0.0334 (3)	
C16	0.11125 (18)	0.17335 (17)	0.22059 (9)	0.0340 (3)	
H16	0.2254	0.1299	0.2016	0.041*	
C17	0.0328 (2)	0.14715 (19)	0.16063 (10)	0.0388 (4)	
H17	−0.0774	0.2166	0.1701	0.047*	
C18	0.0361 (2)	−0.0074 (2)	0.18643 (10)	0.0457 (4)	
H18	0.1366	−0.0810	0.1612	0.055*	
C19	−0.0996 (3)	−0.0302 (3)	0.16736 (14)	0.0674 (6)	
H19A	−0.0715	−0.1337	0.1622	0.081*	
H19B	−0.1265	0.0247	0.1174	0.081*	
C20	−0.1184 (2)	−0.04641 (19)	0.31608 (11)	0.0461 (4)	
H20A	−0.1596	−0.0010	0.3671	0.055*	
H20B	−0.0945	−0.1510	0.3267	0.055*	
C21	0.1052 (2)	0.1722 (2)	0.07350 (10)	0.0448 (4)	
C22	0.2527 (3)	0.0780 (3)	0.03819 (13)	0.0692 (6)	
H22	0.3084	−0.0056	0.0677	0.083*	
C23	0.3190 (3)	0.1071 (4)	−0.04133 (15)	0.0876 (9)	
H23	0.4179	0.0422	−0.0651	0.105*	
C24	0.2369 (4)	0.2330 (4)	−0.08489 (13)	0.0836 (9)	
H24	0.2827	0.2553	−0.1372	0.100*	
C25	0.0906 (4)	0.3232 (3)	−0.05139 (13)	0.0744 (7)	
H25	0.0343	0.4057	−0.0815	0.089*	
C26	0.0235 (3)	0.2939 (2)	0.02735 (12)	0.0577 (5)	
H26	−0.0778	0.3567	0.0495	0.069*	
C27	0.05947 (19)	0.33716 (18)	0.23011 (9)	0.0367 (3)	
C28	0.1557 (2)	0.38602 (19)	0.26347 (10)	0.0404 (4)	
C29	0.3002 (2)	0.3015 (2)	0.29029 (11)	0.0482 (4)	
H29	0.3551	0.1940	0.2924	0.058*	
C30	0.3478 (3)	0.4023 (3)	0.31424 (12)	0.0625 (6)	
H30	0.4434	0.3759	0.3348	0.075*	
C31	0.2380 (3)	0.5465 (3)	0.30139 (13)	0.0641 (6)	
H31	0.2437	0.6372	0.3117	0.077*	
C32	0.1191 (2)	0.5381 (2)	0.27024 (12)	0.0514 (5)	
H32	0.0278	0.6219	0.2554	0.062*	
C33	0.5481 (3)	0.4264 (4)	0.13366 (17)	0.0858 (9)	
H33	0.6415	0.3976	0.1566	0.103*	
C34	0.4395 (3)	0.5697 (3)	0.12193 (16)	0.0783 (7)	
H34	0.4429	0.6592	0.1357	0.094*	
C35	0.3251 (3)	0.5641 (3)	0.08762 (14)	0.0731 (7)	
H35	0.2342	0.6488	0.0731	0.088*	
C36	0.3628 (3)	0.4159 (3)	0.07703 (13)	0.0780 (7)	
H36	0.3038	0.3786	0.0537	0.094*	
C37	0.5015 (4)	0.3312 (3)	0.10598 (16)	0.0880 (9)	
H37	0.5564	0.2238	0.1064	0.106*	
N1	0.33877 (16)	−0.09781 (15)	0.30982 (8)	0.0417 (3)	
N2	0.27266 (17)	−0.00148 (15)	0.46852 (9)	0.0401 (3)	
N3	0.02092 (17)	−0.02272 (15)	0.27455 (8)	0.0394 (3)	
O1	−0.06196 (15)	0.42653 (13)	0.21078 (8)	0.0495 (3)	
S1	−0.26219 (7)	0.03658 (7)	0.25112 (4)	0.06921 (17)	
Fe1	0.33143 (3)	0.44503 (3)	0.196123 (14)	0.04346 (9)	

### Atomic displacement parameters (Å^2^)

**Table:**

	*U*^11^	*U*^22^	*U*^33^	*U*^12^	*U*^13^	*U*^23^
C1	0.0456 (10)	0.0463 (10)	0.0607 (12)	−0.0121 (8)	0.0018 (9)	0.0105 (9)
C2	0.0350 (9)	0.0459 (10)	0.0946 (18)	−0.0132 (8)	−0.0084 (10)	0.0232 (11)
C3	0.0469 (11)	0.0478 (11)	0.0984 (18)	−0.0225 (9)	−0.0349 (12)	0.0216 (11)
C4	0.0543 (11)	0.0437 (10)	0.0737 (14)	−0.0238 (9)	−0.0338 (10)	0.0131 (9)
C5	0.0404 (9)	0.0341 (8)	0.0538 (10)	−0.0190 (7)	−0.0178 (8)	0.0099 (7)
C6	0.0378 (8)	0.0358 (8)	0.0475 (10)	−0.0152 (7)	−0.0062 (7)	0.0112 (7)
C7	0.0382 (8)	0.0312 (7)	0.0313 (8)	−0.0154 (6)	−0.0078 (6)	0.0051 (6)
C8	0.0390 (8)	0.0310 (7)	0.0325 (8)	−0.0167 (6)	−0.0103 (6)	0.0036 (6)
C9	0.0406 (8)	0.0313 (7)	0.0321 (8)	−0.0168 (6)	−0.0086 (6)	0.0020 (6)
C10	0.0538 (10)	0.0394 (8)	0.0350 (9)	−0.0182 (8)	−0.0110 (7)	−0.0032 (7)
C11	0.0531 (10)	0.0382 (9)	0.0420 (10)	−0.0147 (8)	−0.0006 (8)	−0.0078 (7)
C12	0.0384 (9)	0.0347 (8)	0.0515 (10)	−0.0110 (7)	−0.0036 (7)	0.0002 (7)
C13	0.0386 (8)	0.0371 (8)	0.0407 (9)	−0.0155 (7)	−0.0114 (7)	0.0032 (7)
C14	0.0369 (8)	0.0311 (7)	0.0316 (8)	−0.0161 (6)	−0.0071 (6)	0.0026 (6)
C15	0.0384 (8)	0.0333 (7)	0.0294 (8)	−0.0151 (6)	−0.0093 (6)	0.0014 (6)
C16	0.0370 (8)	0.0374 (8)	0.0274 (7)	−0.0145 (6)	−0.0092 (6)	0.0020 (6)
C17	0.0435 (9)	0.0437 (9)	0.0313 (8)	−0.0173 (7)	−0.0131 (7)	0.0002 (7)
C18	0.0602 (11)	0.0469 (9)	0.0354 (9)	−0.0238 (8)	−0.0137 (8)	−0.0041 (7)
C19	0.0998 (18)	0.0735 (14)	0.0591 (13)	−0.0558 (14)	−0.0365 (12)	0.0064 (11)
C20	0.0576 (11)	0.0416 (9)	0.0473 (10)	−0.0280 (8)	−0.0139 (8)	0.0032 (7)
C21	0.0552 (10)	0.0562 (10)	0.0315 (8)	−0.0280 (9)	−0.0146 (7)	0.0000 (7)
C22	0.0605 (13)	0.0970 (18)	0.0449 (11)	−0.0286 (12)	−0.0083 (10)	−0.0011 (11)
C23	0.0710 (16)	0.147 (3)	0.0508 (14)	−0.0541 (18)	0.0067 (12)	−0.0220 (16)
C24	0.121 (2)	0.142 (3)	0.0294 (10)	−0.097 (2)	−0.0126 (13)	0.0053 (13)
C25	0.119 (2)	0.0871 (17)	0.0395 (11)	−0.0624 (17)	−0.0277 (13)	0.0150 (11)
C26	0.0821 (14)	0.0600 (12)	0.0383 (10)	−0.0326 (11)	−0.0224 (10)	0.0058 (8)
C27	0.0435 (9)	0.0397 (8)	0.0263 (7)	−0.0192 (7)	−0.0054 (6)	0.0042 (6)
C28	0.0484 (9)	0.0463 (9)	0.0284 (8)	−0.0240 (8)	−0.0024 (7)	−0.0012 (7)
C29	0.0597 (11)	0.0600 (11)	0.0334 (9)	−0.0336 (9)	−0.0144 (8)	0.0095 (8)
C30	0.0775 (14)	0.0941 (17)	0.0397 (10)	−0.0562 (14)	−0.0179 (10)	0.0025 (10)
C31	0.0844 (15)	0.0754 (15)	0.0500 (12)	−0.0502 (13)	−0.0013 (11)	−0.0187 (10)
C32	0.0582 (11)	0.0512 (10)	0.0458 (10)	−0.0279 (9)	0.0060 (8)	−0.0137 (8)
C33	0.0485 (13)	0.126 (2)	0.0663 (16)	−0.0326 (15)	−0.0005 (11)	0.0155 (16)
C34	0.0784 (16)	0.0964 (19)	0.0698 (16)	−0.0583 (15)	−0.0095 (13)	0.0252 (14)
C35	0.0673 (14)	0.0923 (18)	0.0504 (12)	−0.0342 (13)	−0.0102 (11)	0.0242 (12)
C36	0.0905 (18)	0.117 (2)	0.0314 (11)	−0.0542 (17)	0.0042 (11)	−0.0071 (12)
C37	0.0821 (18)	0.0842 (18)	0.0553 (14)	−0.0089 (15)	0.0164 (13)	−0.0049 (13)
N1	0.0421 (8)	0.0387 (7)	0.0365 (7)	−0.0127 (6)	−0.0044 (6)	0.0035 (6)
N2	0.0459 (8)	0.0376 (7)	0.0425 (8)	−0.0185 (6)	−0.0194 (6)	0.0040 (6)
N3	0.0497 (8)	0.0385 (7)	0.0350 (7)	−0.0218 (6)	−0.0107 (6)	−0.0003 (6)
O1	0.0520 (7)	0.0401 (6)	0.0531 (8)	−0.0135 (6)	−0.0196 (6)	0.0068 (5)
S1	0.0633 (3)	0.0773 (4)	0.0855 (4)	−0.0431 (3)	−0.0344 (3)	0.0174 (3)
Fe1	0.04689 (16)	0.05054 (16)	0.03439 (14)	−0.02474 (12)	−0.00386 (10)	0.00155 (10)

### Geometric parameters (Å, º)

**Table:**

C1—C2	1.367 (3)	C20—H20A	0.9700
C1—C6	1.412 (2)	C20—H20B	0.9700
C1—H1	0.9300	C21—C22	1.377 (3)
C2—C3	1.399 (3)	C21—C26	1.385 (3)
C2—H2	0.9300	C22—C23	1.392 (3)
C3—C4	1.363 (3)	C22—H22	0.9300
C3—H3	0.9300	C23—C24	1.386 (4)
C4—C5	1.414 (2)	C23—H23	0.9300
C4—H4	0.9300	C24—C25	1.349 (4)
C5—N2	1.374 (2)	C24—H24	0.9300
C5—C6	1.417 (3)	C25—C26	1.384 (3)
C6—N1	1.380 (2)	C25—H25	0.9300
C7—N1	1.301 (2)	C26—H26	0.9300
C7—C8	1.421 (2)	C27—O1	1.219 (2)
C7—C15	1.524 (2)	C27—C28	1.467 (2)
C8—N2	1.309 (2)	C28—C32	1.432 (2)
C8—C9	1.463 (2)	C28—C29	1.432 (3)
C9—C10	1.386 (2)	C28—Fe1	2.0342 (17)
C9—C14	1.399 (2)	C29—C30	1.420 (3)
C10—C11	1.387 (3)	C29—Fe1	2.0277 (18)
C10—H10	0.9300	C29—H29	0.9800
C11—C12	1.382 (3)	C30—C31	1.408 (3)
C11—H11	0.9300	C30—Fe1	2.034 (2)
C12—C13	1.385 (2)	C30—H30	0.9800
C12—H12	0.9300	C31—C32	1.410 (3)
C13—C14	1.390 (2)	C31—Fe1	2.036 (2)
C13—H13	0.9300	C31—H31	0.9800
C14—C15	1.526 (2)	C32—Fe1	2.0323 (19)
C15—N3	1.478 (2)	C32—H32	0.9800
C15—C16	1.583 (2)	C33—C37	1.394 (4)
C16—C27	1.528 (2)	C33—C34	1.394 (4)
C16—C17	1.529 (2)	C33—Fe1	2.027 (2)
C16—H16	0.9800	C33—H33	0.9800
C17—C21	1.510 (2)	C34—C35	1.391 (4)
C17—C18	1.527 (2)	C34—Fe1	2.034 (2)
C17—H17	0.9800	C34—H34	0.9800
C18—N3	1.472 (2)	C35—C36	1.405 (4)
C18—C19	1.527 (3)	C35—Fe1	2.038 (2)
C18—H18	0.9800	C35—H35	0.9800
C19—S1	1.807 (3)	C36—C37	1.406 (4)
C19—H19A	0.9700	C36—Fe1	2.044 (2)
C19—H19B	0.9700	C36—H36	0.9800
C20—N3	1.439 (2)	C37—Fe1	2.029 (2)
C20—S1	1.8261 (19)	C37—H37	0.9800
			
C2—C1—C6	120.1 (2)	C30—C29—C28	107.23 (18)
C2—C1—H1	120.0	C30—C29—Fe1	69.79 (11)
C6—C1—H1	120.0	C28—C29—Fe1	69.60 (10)
C1—C2—C3	120.74 (19)	C30—C29—H29	126.4
C1—C2—H2	119.6	C28—C29—H29	126.4
C3—C2—H2	119.6	Fe1—C29—H29	126.4
C4—C3—C2	120.77 (19)	C31—C30—C29	108.91 (19)
C4—C3—H3	119.6	C31—C30—Fe1	69.82 (12)
C2—C3—H3	119.6	C29—C30—Fe1	69.30 (11)
C3—C4—C5	120.1 (2)	C31—C30—H30	125.5
C3—C4—H4	120.0	C29—C30—H30	125.5
C5—C4—H4	120.0	Fe1—C30—H30	125.5
N2—C5—C4	118.66 (17)	C30—C31—C32	108.25 (18)
N2—C5—C6	122.12 (15)	C30—C31—Fe1	69.71 (12)
C4—C5—C6	119.22 (17)	C32—C31—Fe1	69.60 (11)
N1—C6—C1	119.07 (18)	C30—C31—H31	125.9
N1—C6—C5	121.80 (15)	C32—C31—H31	125.9
C1—C6—C5	119.13 (17)	Fe1—C31—H31	125.9
N1—C7—C8	124.02 (15)	C31—C32—C28	108.11 (19)
N1—C7—C15	125.16 (14)	C31—C32—Fe1	69.85 (12)
C8—C7—C15	110.83 (13)	C28—C32—Fe1	69.45 (10)
N2—C8—C7	123.69 (15)	C31—C32—H32	125.9
N2—C8—C9	128.19 (15)	C28—C32—H32	125.9
C7—C8—C9	108.13 (13)	Fe1—C32—H32	125.9
C10—C9—C14	121.27 (15)	C37—C33—C34	107.9 (3)
C10—C9—C8	130.11 (15)	C37—C33—Fe1	69.99 (15)
C14—C9—C8	108.62 (13)	C34—C33—Fe1	70.20 (13)
C9—C10—C11	118.26 (16)	C37—C33—H33	126.0
C9—C10—H10	120.9	C34—C33—H33	126.0
C11—C10—H10	120.9	Fe1—C33—H33	126.0
C12—C11—C10	120.69 (16)	C35—C34—C33	108.4 (3)
C12—C11—H11	119.7	C35—C34—Fe1	70.16 (13)
C10—C11—H11	119.7	C33—C34—Fe1	69.63 (14)
C11—C12—C13	121.34 (16)	C35—C34—H34	125.8
C11—C12—H12	119.3	C33—C34—H34	125.8
C13—C12—H12	119.3	Fe1—C34—H34	125.8
C12—C13—C14	118.59 (16)	C34—C35—C36	108.2 (2)
C12—C13—H13	120.7	C34—C35—Fe1	69.88 (13)
C14—C13—H13	120.7	C36—C35—Fe1	70.09 (13)
C13—C14—C9	119.83 (15)	C34—C35—H35	125.9
C13—C14—C15	128.71 (14)	C36—C35—H35	125.9
C9—C14—C15	111.46 (13)	Fe1—C35—H35	125.9
N3—C15—C7	109.71 (12)	C35—C36—C37	107.2 (3)
N3—C15—C14	116.40 (13)	C35—C36—Fe1	69.62 (13)
C7—C15—C14	100.90 (12)	C37—C36—Fe1	69.25 (14)
N3—C15—C16	104.60 (12)	C35—C36—H36	126.4
C7—C15—C16	110.87 (12)	C37—C36—H36	126.4
C14—C15—C16	114.42 (12)	Fe1—C36—H36	126.4
C27—C16—C17	112.14 (13)	C33—C37—C36	108.3 (3)
C27—C16—C15	113.69 (12)	C33—C37—Fe1	69.79 (15)
C17—C16—C15	104.07 (12)	C36—C37—Fe1	70.36 (14)
C27—C16—H16	108.9	C33—C37—H37	125.8
C17—C16—H16	108.9	C36—C37—H37	125.8
C15—C16—H16	108.9	Fe1—C37—H37	125.8
C21—C17—C18	115.79 (14)	C7—N1—C6	114.22 (14)
C21—C17—C16	113.64 (14)	C8—N2—C5	114.15 (14)
C18—C17—C16	103.98 (13)	C20—N3—C18	112.11 (14)
C21—C17—H17	107.7	C20—N3—C15	121.31 (13)
C18—C17—H17	107.7	C18—N3—C15	110.92 (13)
C16—C17—H17	107.7	C19—S1—C20	86.86 (10)
N3—C18—C17	105.17 (13)	C33—Fe1—C29	122.00 (10)
N3—C18—C19	108.33 (16)	C33—Fe1—C37	40.22 (13)
C17—C18—C19	113.38 (16)	C29—Fe1—C37	109.09 (10)
N3—C18—H18	109.9	C33—Fe1—C32	156.60 (12)
C17—C18—H18	109.9	C29—Fe1—C32	69.34 (8)
C19—C18—H18	109.9	C37—Fe1—C32	161.49 (12)
C18—C19—S1	105.74 (14)	C33—Fe1—C34	40.17 (11)
C18—C19—H19A	110.6	C29—Fe1—C34	156.53 (10)
S1—C19—H19A	110.6	C37—Fe1—C34	67.43 (13)
C18—C19—H19B	110.6	C32—Fe1—C34	121.59 (10)
S1—C19—H19B	110.6	C33—Fe1—C30	105.84 (11)
H19A—C19—H19B	108.7	C29—Fe1—C30	40.92 (8)
N3—C20—S1	107.38 (12)	C37—Fe1—C30	123.37 (12)
N3—C20—H20A	110.2	C32—Fe1—C30	68.31 (9)
S1—C20—H20A	110.2	C34—Fe1—C30	119.99 (10)
N3—C20—H20B	110.2	C33—Fe1—C28	159.83 (11)
S1—C20—H20B	110.2	C29—Fe1—C28	41.29 (7)
H20A—C20—H20B	108.5	C37—Fe1—C28	125.45 (11)
C22—C21—C26	118.36 (19)	C32—Fe1—C28	41.24 (7)
C22—C21—C17	121.84 (17)	C34—Fe1—C28	159.53 (10)
C26—C21—C17	119.79 (17)	C30—Fe1—C28	68.71 (8)
C21—C22—C23	120.6 (2)	C33—Fe1—C31	120.41 (12)
C21—C22—H22	119.7	C29—Fe1—C31	68.97 (9)
C23—C22—H22	119.7	C37—Fe1—C31	157.48 (13)
C24—C23—C22	119.7 (3)	C32—Fe1—C31	40.55 (9)
C24—C23—H23	120.2	C34—Fe1—C31	104.96 (11)
C22—C23—H23	120.2	C30—Fe1—C31	40.47 (10)
C25—C24—C23	119.9 (2)	C28—Fe1—C31	68.85 (8)
C25—C24—H24	120.0	C33—Fe1—C35	67.53 (11)
C23—C24—H24	120.0	C29—Fe1—C35	162.36 (10)
C24—C25—C26	120.5 (2)	C37—Fe1—C35	67.60 (11)
C24—C25—H25	119.7	C32—Fe1—C35	107.97 (10)
C26—C25—H25	119.7	C34—Fe1—C35	39.96 (10)
C25—C26—C21	120.8 (2)	C30—Fe1—C35	155.73 (11)
C25—C26—H26	119.6	C28—Fe1—C35	125.32 (9)
C21—C26—H26	119.6	C31—Fe1—C35	121.05 (11)
O1—C27—C28	120.24 (15)	C33—Fe1—C36	67.80 (12)
O1—C27—C16	120.13 (15)	C29—Fe1—C36	125.97 (10)
C28—C27—C16	119.63 (14)	C37—Fe1—C36	40.40 (11)
C32—C28—C29	107.48 (16)	C32—Fe1—C36	124.65 (10)
C32—C28—C27	122.76 (17)	C34—Fe1—C36	67.51 (12)
C29—C28—C27	129.68 (16)	C30—Fe1—C36	161.01 (12)
C32—C28—Fe1	69.31 (10)	C28—Fe1—C36	110.71 (9)
C29—C28—Fe1	69.11 (10)	C31—Fe1—C36	158.33 (12)
C27—C28—Fe1	124.33 (11)	C35—Fe1—C36	40.28 (11)
			
C6—C1—C2—C3	0.7 (3)	C28—C29—Fe1—C33	−164.98 (15)
C1—C2—C3—C4	0.2 (3)	C30—C29—Fe1—C37	119.30 (17)
C2—C3—C4—C5	−0.8 (3)	C28—C29—Fe1—C37	−122.42 (15)
C3—C4—C5—N2	−179.93 (16)	C30—C29—Fe1—C32	−80.33 (14)
C3—C4—C5—C6	0.5 (3)	C28—C29—Fe1—C32	37.95 (11)
C2—C1—C6—N1	179.02 (17)	C30—C29—Fe1—C34	42.2 (3)
C2—C1—C6—C5	−0.9 (3)	C28—C29—Fe1—C34	160.5 (2)
N2—C5—C6—N1	0.8 (2)	C28—C29—Fe1—C30	118.28 (18)
C4—C5—C6—N1	−179.63 (15)	C30—C29—Fe1—C28	−118.28 (18)
N2—C5—C6—C1	−179.19 (16)	C30—C29—Fe1—C31	−36.80 (14)
C4—C5—C6—C1	0.3 (2)	C28—C29—Fe1—C31	81.48 (12)
N1—C7—C8—N2	0.0 (2)	C30—C29—Fe1—C35	−164.7 (3)
C15—C7—C8—N2	−179.74 (14)	C28—C29—Fe1—C35	−46.4 (4)
N1—C7—C8—C9	−179.46 (14)	C30—C29—Fe1—C36	161.17 (15)
C15—C7—C8—C9	0.77 (17)	C28—C29—Fe1—C36	−80.55 (15)
N2—C8—C9—C10	0.8 (3)	C36—C37—Fe1—C33	−119.1 (3)
C7—C8—C9—C10	−179.72 (16)	C33—C37—Fe1—C29	−117.33 (17)
N2—C8—C9—C14	−178.45 (15)	C36—C37—Fe1—C29	123.54 (17)
C7—C8—C9—C14	1.02 (17)	C33—C37—Fe1—C32	160.9 (3)
C14—C9—C10—C11	1.3 (2)	C36—C37—Fe1—C32	41.7 (4)
C8—C9—C10—C11	−177.85 (16)	C33—C37—Fe1—C34	37.81 (17)
C9—C10—C11—C12	−0.5 (3)	C36—C37—Fe1—C34	−81.33 (18)
C10—C11—C12—C13	−0.8 (3)	C33—C37—Fe1—C30	−74.2 (2)
C11—C12—C13—C14	1.2 (2)	C36—C37—Fe1—C30	166.68 (15)
C12—C13—C14—C9	−0.4 (2)	C33—C37—Fe1—C28	−160.48 (15)
C12—C13—C14—C15	−179.37 (15)	C36—C37—Fe1—C28	80.39 (19)
C10—C9—C14—C13	−0.9 (2)	C33—C37—Fe1—C31	−36.4 (4)
C8—C9—C14—C13	178.41 (14)	C36—C37—Fe1—C31	−155.5 (2)
C10—C9—C14—C15	178.23 (14)	C33—C37—Fe1—C35	81.21 (18)
C8—C9—C14—C15	−2.42 (17)	C36—C37—Fe1—C35	−37.92 (17)
N1—C7—C15—N3	54.8 (2)	C33—C37—Fe1—C36	119.1 (3)
C8—C7—C15—N3	−125.41 (14)	C31—C32—Fe1—C33	−42.3 (3)
N1—C7—C15—C14	178.19 (15)	C28—C32—Fe1—C33	−161.7 (2)
C8—C7—C15—C14	−2.04 (16)	C31—C32—Fe1—C29	81.42 (14)
N1—C7—C15—C16	−60.2 (2)	C28—C32—Fe1—C29	−38.00 (11)
C8—C7—C15—C16	119.56 (14)	C31—C32—Fe1—C37	170.0 (3)
C13—C14—C15—N3	−59.6 (2)	C28—C32—Fe1—C37	50.5 (4)
C9—C14—C15—N3	121.33 (14)	C31—C32—Fe1—C34	−75.36 (17)
C13—C14—C15—C7	−178.22 (15)	C28—C32—Fe1—C34	165.22 (13)
C9—C14—C15—C7	2.71 (16)	C31—C32—Fe1—C30	37.41 (14)
C13—C14—C15—C16	62.7 (2)	C28—C32—Fe1—C30	−82.01 (12)
C9—C14—C15—C16	−116.37 (14)	C31—C32—Fe1—C28	119.42 (18)
N3—C15—C16—C27	142.11 (13)	C28—C32—Fe1—C31	−119.42 (18)
C7—C15—C16—C27	−99.71 (15)	C31—C32—Fe1—C35	−117.07 (16)
C14—C15—C16—C27	13.59 (18)	C28—C32—Fe1—C35	123.51 (13)
N3—C15—C16—C17	19.83 (15)	C31—C32—Fe1—C36	−158.42 (15)
C7—C15—C16—C17	138.01 (13)	C28—C32—Fe1—C36	82.16 (15)
C14—C15—C16—C17	−108.70 (14)	C35—C34—Fe1—C33	119.4 (3)
C27—C16—C17—C21	78.01 (18)	C35—C34—Fe1—C29	167.7 (2)
C15—C16—C17—C21	−158.68 (14)	C33—C34—Fe1—C29	48.2 (4)
C27—C16—C17—C18	−155.23 (14)	C35—C34—Fe1—C37	81.59 (19)
C15—C16—C17—C18	−31.93 (16)	C33—C34—Fe1—C37	−37.85 (18)
C21—C17—C18—N3	157.97 (14)	C35—C34—Fe1—C32	−80.21 (19)
C16—C17—C18—N3	32.57 (17)	C33—C34—Fe1—C32	160.35 (18)
C21—C17—C18—C19	−83.9 (2)	C35—C34—Fe1—C30	−161.81 (16)
C16—C17—C18—C19	150.75 (16)	C33—C34—Fe1—C30	78.7 (2)
N3—C18—C19—S1	31.37 (19)	C35—C34—Fe1—C28	−51.5 (4)
C17—C18—C19—S1	−84.96 (17)	C33—C34—Fe1—C28	−170.9 (2)
C18—C17—C21—C22	−50.0 (2)	C35—C34—Fe1—C31	−120.83 (18)
C16—C17—C21—C22	70.3 (2)	C33—C34—Fe1—C31	119.7 (2)
C18—C17—C21—C26	130.92 (18)	C33—C34—Fe1—C35	−119.4 (3)
C16—C17—C21—C26	−108.79 (19)	C35—C34—Fe1—C36	37.68 (17)
C26—C21—C22—C23	1.6 (3)	C33—C34—Fe1—C36	−81.8 (2)
C17—C21—C22—C23	−177.4 (2)	C31—C30—Fe1—C33	118.56 (16)
C21—C22—C23—C24	1.0 (4)	C29—C30—Fe1—C33	−120.90 (16)
C22—C23—C24—C25	−3.0 (4)	C31—C30—Fe1—C29	−120.54 (19)
C23—C24—C25—C26	2.3 (4)	C31—C30—Fe1—C37	158.79 (16)
C24—C25—C26—C21	0.4 (4)	C29—C30—Fe1—C37	−80.68 (18)
C22—C21—C26—C25	−2.3 (3)	C31—C30—Fe1—C32	−37.48 (12)
C17—C21—C26—C25	176.74 (19)	C29—C30—Fe1—C32	83.06 (13)
C17—C16—C27—O1	20.1 (2)	C31—C30—Fe1—C34	77.45 (17)
C15—C16—C27—O1	−97.61 (17)	C29—C30—Fe1—C34	−162.02 (14)
C17—C16—C27—C28	−160.58 (14)	C31—C30—Fe1—C28	−81.95 (13)
C15—C16—C27—C28	81.70 (17)	C29—C30—Fe1—C28	38.59 (12)
O1—C27—C28—C32	−4.0 (2)	C29—C30—Fe1—C31	120.54 (19)
C16—C27—C28—C32	176.64 (15)	C31—C30—Fe1—C35	48.3 (3)
O1—C27—C28—C29	179.61 (17)	C29—C30—Fe1—C35	168.8 (2)
C16—C27—C28—C29	0.3 (3)	C31—C30—Fe1—C36	−173.9 (3)
O1—C27—C28—Fe1	−90.07 (18)	C29—C30—Fe1—C36	−53.4 (3)
C16—C27—C28—Fe1	90.62 (17)	C32—C28—Fe1—C33	158.8 (3)
C32—C28—C29—C30	1.0 (2)	C29—C28—Fe1—C33	39.6 (3)
C27—C28—C29—C30	177.79 (17)	C27—C28—Fe1—C33	−85.0 (3)
Fe1—C28—C29—C30	59.91 (13)	C32—C28—Fe1—C29	119.19 (16)
C32—C28—C29—Fe1	−58.89 (12)	C27—C28—Fe1—C29	−124.54 (19)
C27—C28—C29—Fe1	117.88 (17)	C32—C28—Fe1—C37	−162.49 (15)
C28—C29—C30—C31	−1.1 (2)	C29—C28—Fe1—C37	78.32 (16)
Fe1—C29—C30—C31	58.71 (15)	C27—C28—Fe1—C37	−46.2 (2)
C28—C29—C30—Fe1	−59.79 (12)	C29—C28—Fe1—C32	−119.19 (16)
C29—C30—C31—C32	0.7 (2)	C27—C28—Fe1—C32	116.26 (19)
Fe1—C30—C31—C32	59.11 (14)	C32—C28—Fe1—C34	−38.4 (3)
C29—C30—C31—Fe1	−58.39 (14)	C29—C28—Fe1—C34	−157.6 (3)
C30—C31—C32—C28	−0.1 (2)	C27—C28—Fe1—C34	77.8 (3)
Fe1—C31—C32—C28	59.11 (13)	C32—C28—Fe1—C30	80.95 (13)
C30—C31—C32—Fe1	−59.18 (15)	C29—C28—Fe1—C30	−38.24 (12)
C29—C28—C32—C31	−0.6 (2)	C27—C28—Fe1—C30	−162.79 (17)
C27—C28—C32—C31	−177.64 (16)	C32—C28—Fe1—C31	37.39 (13)
Fe1—C28—C32—C31	−59.36 (14)	C29—C28—Fe1—C31	−81.81 (13)
C29—C28—C32—Fe1	58.77 (12)	C27—C28—Fe1—C31	153.65 (17)
C27—C28—C32—Fe1	−118.28 (15)	C32—C28—Fe1—C35	−76.42 (16)
C37—C33—C34—C35	0.4 (3)	C29—C28—Fe1—C35	164.38 (14)
Fe1—C33—C34—C35	−59.68 (17)	C27—C28—Fe1—C35	39.8 (2)
C37—C33—C34—Fe1	60.08 (18)	C32—C28—Fe1—C36	−119.40 (14)
C33—C34—C35—C36	−0.5 (3)	C29—C28—Fe1—C36	121.41 (14)
Fe1—C34—C35—C36	−59.85 (16)	C27—C28—Fe1—C36	−3.13 (18)
C33—C34—C35—Fe1	59.35 (17)	C30—C31—Fe1—C33	−78.45 (16)
C34—C35—C36—C37	0.4 (3)	C32—C31—Fe1—C33	161.96 (14)
Fe1—C35—C36—C37	−59.32 (16)	C30—C31—Fe1—C29	37.18 (12)
C34—C35—C36—Fe1	59.72 (17)	C32—C31—Fe1—C29	−82.41 (13)
C34—C33—C37—C36	−0.2 (3)	C30—C31—Fe1—C37	−52.1 (3)
Fe1—C33—C37—C36	60.06 (17)	C32—C31—Fe1—C37	−171.7 (3)
C34—C33—C37—Fe1	−60.21 (17)	C30—C31—Fe1—C32	119.59 (18)
C35—C36—C37—C33	−0.1 (3)	C30—C31—Fe1—C34	−118.95 (14)
Fe1—C36—C37—C33	−59.71 (17)	C32—C31—Fe1—C34	121.46 (14)
C35—C36—C37—Fe1	59.56 (16)	C32—C31—Fe1—C30	−119.59 (18)
C8—C7—N1—C6	0.6 (2)	C30—C31—Fe1—C28	81.59 (13)
C15—C7—N1—C6	−179.67 (14)	C32—C31—Fe1—C28	−38.00 (12)
C1—C6—N1—C7	179.03 (15)	C30—C31—Fe1—C35	−159.02 (13)
C5—C6—N1—C7	−1.0 (2)	C32—C31—Fe1—C35	81.39 (15)
C7—C8—N2—C5	−0.3 (2)	C30—C31—Fe1—C36	174.6 (2)
C9—C8—N2—C5	179.13 (14)	C32—C31—Fe1—C36	55.0 (3)
C4—C5—N2—C8	−179.70 (15)	C34—C35—Fe1—C33	−37.44 (19)
C6—C5—N2—C8	−0.2 (2)	C36—C35—Fe1—C33	81.69 (19)
S1—C20—N3—C18	−26.58 (17)	C34—C35—Fe1—C29	−163.7 (3)
S1—C20—N3—C15	107.71 (14)	C36—C35—Fe1—C29	−44.6 (4)
C17—C18—N3—C20	118.54 (15)	C34—C35—Fe1—C37	−81.1 (2)
C19—C18—N3—C20	−3.0 (2)	C36—C35—Fe1—C37	38.02 (17)
C17—C18—N3—C15	−20.56 (18)	C34—C35—Fe1—C32	118.06 (17)
C19—C18—N3—C15	−142.10 (16)	C36—C35—Fe1—C32	−122.81 (15)
C7—C15—N3—C20	106.63 (16)	C36—C35—Fe1—C34	119.1 (2)
C14—C15—N3—C20	−7.1 (2)	C34—C35—Fe1—C30	41.1 (3)
C16—C15—N3—C20	−134.40 (15)	C36—C35—Fe1—C30	160.2 (2)
C7—C15—N3—C18	−118.60 (14)	C34—C35—Fe1—C28	160.41 (16)
C14—C15—N3—C18	127.69 (15)	C36—C35—Fe1—C28	−80.46 (17)
C16—C15—N3—C18	0.37 (17)	C34—C35—Fe1—C31	75.55 (19)
C18—C19—S1—C20	−39.25 (15)	C36—C35—Fe1—C31	−165.32 (15)
N3—C20—S1—C19	38.40 (13)	C34—C35—Fe1—C36	−119.1 (2)
C37—C33—Fe1—C29	81.85 (19)	C35—C36—Fe1—C33	−80.97 (18)
C34—C33—Fe1—C29	−159.50 (16)	C37—C36—Fe1—C33	37.53 (18)
C34—C33—Fe1—C37	118.7 (3)	C35—C36—Fe1—C29	164.76 (14)
C37—C33—Fe1—C32	−164.8 (2)	C37—C36—Fe1—C29	−76.7 (2)
C34—C33—Fe1—C32	−46.1 (3)	C35—C36—Fe1—C37	−118.5 (2)
C37—C33—Fe1—C34	−118.7 (3)	C35—C36—Fe1—C32	76.38 (17)
C37—C33—Fe1—C30	123.35 (17)	C37—C36—Fe1—C32	−165.11 (17)
C34—C33—Fe1—C30	−118.00 (18)	C35—C36—Fe1—C34	−37.39 (15)
C37—C33—Fe1—C28	52.1 (4)	C37—C36—Fe1—C34	81.1 (2)
C34—C33—Fe1—C28	170.8 (2)	C35—C36—Fe1—C30	−154.7 (3)
C37—C33—Fe1—C31	164.73 (16)	C37—C36—Fe1—C30	−36.2 (4)
C34—C33—Fe1—C31	−76.6 (2)	C35—C36—Fe1—C28	120.66 (15)
C37—C33—Fe1—C35	−81.40 (19)	C37—C36—Fe1—C28	−120.84 (18)
C34—C33—Fe1—C35	37.25 (18)	C35—C36—Fe1—C31	36.0 (3)
C37—C33—Fe1—C36	−37.69 (17)	C37—C36—Fe1—C31	154.5 (3)
C34—C33—Fe1—C36	80.96 (19)	C37—C36—Fe1—C35	118.5 (2)
C30—C29—Fe1—C33	76.74 (19)		

### Hydrogen-bond geometry (Å, º)

**Table:**

*D*—H···*A*	*D*—H	H···*A*	*D*···*A*	*D*—H···*A*
C25—H25···O1^i^	0.93	2.58	3.371 (3)	143

Symmetry code: (i) −*x*, −*y*+1, −*z*.
